# Interaction of prenatal maternal smoking, interleukin 13 genetic variants and DNA methylation influencing airflow and airway reactivity

**DOI:** 10.1186/1868-7083-5-22

**Published:** 2013-12-06

**Authors:** Veeresh K Patil, John W Holloway, Hongmei Zhang, Nelis Soto-Ramirez, Susan Ewart, S Hasan Arshad, Wilfried Karmaus

**Affiliations:** 1 David Hide Asthma and Allergy Research Centre, St Mary’s Hospital, Newport, Isle of Wight PO30 5TG, UK; 2 Clinical and Experimental Sciences, Faculty of Medicine, University of Southampton, Southampton, UK; 3 Human Development and Health, Faculty of Medicine, University of Southampton, Southampton, UK; 4 Division of Epidemiology, Biostatistics, & Environmental Health, School of Public Health, University of Memphis, Memphis, TN, USA; 5 Department of Large Animal Clinical Sciences, Michigan State University, East Lansing, MI, USA

**Keywords:** Asthma genetics and epigenetics, Airway reactivity, DNA methylation, IL13 gene, Lung functions, Maternal smoking during pregnancy

## Abstract

**Background:**

Asthma is characterized by airflow limitation and airway reactivity (AR). Interleukin-13 (IL-13) is involved in the pathogenesis of asthma. Two functional SNPs, rs20541 and rs1800925, of the IL-13 gene (*IL13*) have been frequently associated with asthma-related lung functions. However, genetic variation alone does not fully explain asthma risk. DNA-methylation (DNA-M) is an epigenetic mechanism that regulates gene expression and can be influenced by both environment and genetic variants. To explore the interplay of prenatal maternal smoking, genetic variants and DNA-M, we used a two-stage model: (1) identifying cytosine phosphate guanine (CpG) sites where DNA-M is influenced by the interaction between genetic variants and maternal smoking during pregnancy (conditional *methQTL* (methylation quantitative trait loci)); and (2) determining the effect of the interaction between DNA-M of CpG (from stage 1) and SNPs (modifying genetic variants; *modGV*) on airflow limitation and AR in 245 female participants of the Isle of Wight birth cohort. DNA-M was assessed using the Illumina Infinium HumanMethylation450 BeadChip.

**Findings:**

Six CpG sites were analyzed in stage 1. DNA-M at cg13566430 was influenced by interaction of maternal smoking during pregnancy and rs20541. In stage 2, genotype at rs1800925 interacted with DNA-M at cg13566430 significantly affecting airflow limitation (*P* = 0.042) and AR (*P* = 0.01).

**Conclusion:**

Both genetic variants and environment affect DNA-M. This study supports the proposed two-stage model (methQTL and modGV) to study genetic variants, environment and DNA-M interactions in asthma-related lung function.

## Findings

Asthma is a chronic airway disease characterized by airflow limitation and airway reactivity (AR) and exhibits wide heterogeneity in disease susceptibly and phenotypes. Multiple genes, gene-gene and gene-environment interactions have been shown to play a role in determining susceptibility to asthma and associated phenotypes of lung function and AR. Maternal smoking during pregnancy is a significant risk factor for developing asthma in offspring [1] and can affect offspring lung function [2]. Interleukin-13 (IL-13) is a recognized effector in airway inflammation, reactivity and remodeling. *IL13* is located on chromosome 5q31, and has been consistently associated with asthma [3,4]. Several functional genetic variants occur in *IL13* including rs1800925 (−1112C/T) and rs20541 (R130Q, +2044A/G). Recent meta-analyses have shown that both SNPs are associated with asthma risk [5,6] and with forced expiratory volume in 1 second (FEV_1_) and FEV_1_/forced vital capacity (FVC) in asthmatics [7,8]. In severe asthma, monoclonal antibody to IL-13 use is associated with improvement in lung function in humans [9] and airway hyper-responsiveness in a murine model [10].

**Figure 1:**
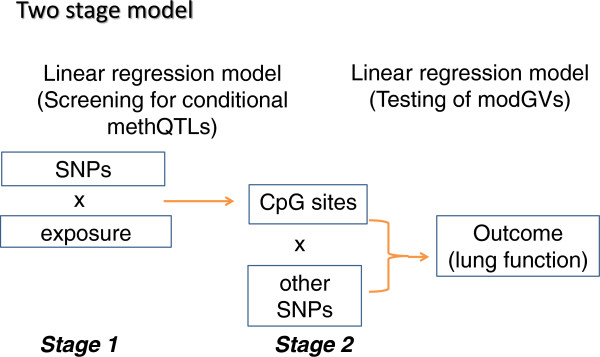
**Two-stage model to investigate environment, SNP and DNA methylation interactions influencing asthma-related lung functions (Modified from Karmaus et al. **[16]**.** CpG, cytosine phosphate guanine; methQTL, methylation, quantitative trait loci (genetic variants that change the susceptibility for DNA methylation); modGV, modifiable genetic variants (genetic variants that are modified by DNA methylation).

DNA-methylation (DNA-M) represents a site of molecular interaction between the environment and genome. There is growing evidence that DNA-M plays a role in complex diseases like asthma [11] and can be modified by environmental exposures such as tobacco smoke [12] as well as by disease-associated genotypes [13]. We have previously shown that *IL13* polymorphism modifies the impact of *in utero* tobacco smoke exposure on childhood asthma, suggesting a role for gene-environment interaction [14]. Recently we have also shown that genetic variants in the IL-4 receptor interact with DNA-M to determine risk of asthma [15]. DNA-M is a potential integrator of different signals affecting disease susceptibility, with both environment and genotype influencing methylation levels. Karmaus and colleagues [16] proposed a two-stage model to incorporate the role of genetic variants, environment and DNA-M interactions in asthma. In stage 1, an environmental exposure and genetic variant interact to influence DNA-M at a specific site in an adjacent locus. This stage identifies the conditional methQTL (methylation quantitative trait loci) and the change in DNA-M once established can differentially regulate gene activity. In stage 2, the phenotypic effects of sequence variants of the gene (modifiable genetic variants (modGVs)) can be modified by the pre-established methylation by the conditional methQTL. This two stage model for asthma-related lung function is depicted in Figure 1. Given our previous observation of the interaction between maternal smoke exposure and genotype in determining asthma, we hypothesize that this interaction would occur through a mechanism involving methQTL and/or modGVs.

### Study population and assessments

The Isle of Wight birth cohort (n = 1,456) was established in 1989 and has been followed up through childhood. At 18 years of age, spirometry and bronchial challenge tests were done according to American Thoracic Society guidelines [17,18]. We analyzed lung function measurements and DNA-M data of 245 females aged 18 years who were randomly selected from the cohort population for epigenetic studies. Comparison of the analyzed sample with whole cohort females in terms of risk factors have been described elsewhere [15]. A continuous measure of AR (dose response slope (DRS)) was used. DRS is the gradient of FEV_1_ drop from baseline with each successive incremental dose of methacholine administered. A transformation of Log_10_ (DRS + 10) was required to satisfy the distributional assumption of normal data. A higher positive value of this transformation signifies greater AR. A description of the 18-year follow-up of the cohort is previously reported [19].

### Genotyping and SNP selection

DNA was isolated from peripheral blood leucocytes collected at age 18. *IL13* polymorphisms were genotyped as described by Sadeghnejad and colleagues [14]. Two known functional SNPs, rs1800925 (n = 234) and rs20541 (n = 235), frequently associated with asthma and related lung functions were used in this analysis. These SNPs were not in linkage disequilibrium in this cohort as described elsewhere [14].

### DNA-methylation

One microgram of DNA was bisulfite-treated using the EZ 96-DNA-methylation kit (Zymo Research, Irvine, CA, USA), following the manufacturer’s protocol. Genome-wide DNA- M was assessed using the Illumina Infinium-HumanMethylation450 BeadChip (Illumina, Inc., Hayward, CA, USA) as described previously [15].

### Statistical methods

The pre-processed DNA-M beta (β) values, presented as the proportion of intensity of methylated (M) over the sum of methylated (M) and unmethylated (U) sites (β = M/[c + M + U] with c being a constant to prevent dividing by zero), were used to estimate the effect of DNA-M [20]. The R-package IMA in Bioconductor (IMA is implemented in the R language and is freely available from http://www.rforge.net/IMA) was used for the pre-processing [21]. SNP genotype-dependent methylation was analyzed using the Kruskal-Wallis test. Interaction was tested using multiple linear regressions. Statistical analyses were performed using IBM SPSS Statistics, Version-19.0 (IBM SPSS Statistics for Windows, Version 19.0. Armonk, NY, USA: IBM Corp,). The statistical significance was set at 0.05.

## Results

**Figure 2:**
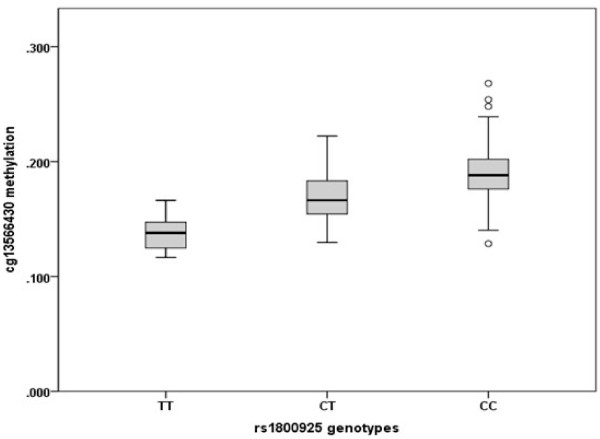
**Whisker plot showing *IL13 *** **rs1800925 genotype-dependant DNA methylation of cg13566430.**

Six cytosine phosphate guanine (CpG) sites were identified in the promoter region of *IL13.* The location and description of the CpG sites and of SNPs are shown in Table 1. All the six CpG sites spanning the promoter region of *IL13* were analyzed in Stage 1. The effect of maternal smoking during pregnancy interacting with both SNPs was explored independently for DNA-M at each CpG site. DNA-M at cg13566430 was influenced by the interaction of rs20541 and maternal smoking during pregnancy (*P = 0.043);* this remained significant after correcting for personal smoking at 18 years (*P = 0.041*). DNA-M at cg13566430 also showed a genotype dependent methylation for rs1800925 (Kruskall Wallis test *P* < 0.001; Figure 2).

**Table 1:** **Location, position and description of rs1800925, rs20541 and CpG sites on the promoter region of *IL13 *** **gene**

**SNP**	**Chromosomal location**	**Position**	**Genotypes**	**n (%)**	
rs1800925	5:131992809	5′ Promoter upstream	TT	8 (3.4%)	
			CT	68 (28.8%)	
			CC	158 (67.8%)	
rs20541	5:131995964	Exon 4	AA	10 (4.3%)	
			AG	66 (28.1%)	
			GG	159 (67.7%)	
**CpG**			**Median**	**Percentiles (5%, 95%)**	
cg13566430	5:131992455	TSS1500	0.18	0.14	0.23
cg04303330	5: 131992430	TSS1500	0.30	0.23	0.36
cg06584121	5: 131993818	TSS200	0.80	0.730	0.84
cg06967316	5: 131993853	TSS200	0.74	0.66	0.80
cg14523284	5: 131993614	TSS1500	0.86	0.83	0.89
cg15329179	5: 131993728	TSS200	0.87	0.81	0.90

CpG, cytosine phosphate guanine; TSS200, 200 base pairs from transcription start site; TSS1500, 1500 base pairs from transcription start site.

**Figure 3:**
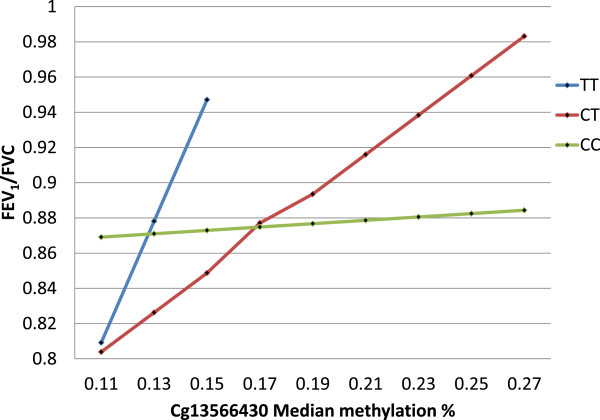
**Graph showing interaction of rs1800925 and methylation at cg13566430 influencing FEV**_**1**_**/FVC.** FEV_1_, forced expiratory volume in 1 second; FVC, forced vital capacity.

In the next step, the interaction between DNA-M at cg13566430 and rs1800925 genotype on lung function (FEV_1_/FVC and DRS) was explored. DNA-M at cg13566430 significantly interacted with rs1800925 genotype to determine FEV_1_/FVC and DRS (Table 2). In subjects with the TT genotype, FEV_1_/FVC increases with increasing methylation of cg13566430 (coefficient 3.274, *P = 0.042*), for the CT genotype group the increase was smaller (coefficient 0.799, *P* = 0.086) and CC was the reference genotype. The graph in Figure 3 provides a visual description of the effect of DNA-M at cg13566430 and rs1800925 on FEV_1_/FVC. DRS decreases quickly with increasing methylation of cg13566430 in TT genotype (coefficient −27.497, *P* = 0.010), while the drop in DRS in CT is insignificant (coefficient −0.809, *P* = 0.742).

**Table 2:** **Interaction of methylation at cg13566430 and rs1800925 genotypes on FEV**_**1**_**/FVC and DRS**

		**FEV**_**1**_**/FVC**			**DRS**		
		**Estimate**	**95% CI**	***P***	**Estimate**	**95% CI**	***P***
**Main effects**							
**cg13566430**		0.175	−0.166 to 0.517	0.313	−0.568	−2.361 to 1.226	0.533
**rs20541**	**AA**	0.020	−0.024 to 0.064	0.369	−0.075	−0.278 to 0.129	0.469
	**AG**	0.006	−0.014 to 0.026	0.548	0.036	−0.076 to 0.149	0.523
	**GG**		Reference			Reference	
**rs1800925**	**TT**	0.030	−0.019 to 0.079	0.231	0.299	0.045 to 0.554	0.021
	**CT**	−0.001	−0.021 to 0.019	−0.001	−0.026	−0.134 to 0.083	0.644
	**CC**		Reference			Reference	
**Interaction**							
**rs1800925**	**TT**	−0.412	−0.855 to 0.030	0.068	3.989	1.194 to 6.785	0.005
	**CT**	−0.134	−0.296 to 0.028	0.106	0.124	−0.733 to 0.982	0.775
	**CC**		Reference			Reference	
**cg13566430**		0.174	−0.301 to 0.648	0.472	0.624	−1.926 to 3.174	0.629
**rs1800925× cg13566430**	**TT**	3.274	0.114 to 6.434	0.042	−27.497	−48.283 to −6.710	0.010
	**CT**	0.799	−0.114 to 1.712	0.086	−0.809	−5.661 to 4.043	0.742
	**CC**		Reference			Reference	

DRS, dose response slope; FEV_1_, forced expiratory volume in 1 second; FVC, forced vital capacity.

## Discussion

This study tested a two-stage model for integrating the interactions of maternal smoking during pregnancy, genetic variants and DNA-M for an asthma candidate gene *IL13*. We show that interaction of a functional *IL13* SNP, rs20541, and maternal smoking during pregnancy influenced DNA-M at cg13566430. We also show that DNA-M at cg13566430 interacts with genotype of another functional SNP, rs1800925, to affect airflow limitation and AR. Michel and colleagues [22] examined the effect of farm exposure on DNA-M of ten asthma candidate genes and found that DNA-M at one *IL13* site (spanning rs1800925; similar to the site in our study) was more methylated in the exposed group compared to the non-exposed group. They did not see significant differential methylation of *IL13* between asthmatic and non-asthmatic children but asthma-related traits were not tested and also the interaction with genetic variants was not examined. Our approach identifies the effect of environmental exposure and genetic variants on DNA-M and then also combines the interaction of other modifiable genetic variants with DNA-M on the outcomes. We show genotype-dependent DNA-M in the *IL13* promoter region; methylation at cg13566430 revealed variation in levels dependent on the genotype of rs1800925, as seen in other genes [23]. This varying distribution of DNA-M across genotypes supports the plausible role for DNA-M in the pathway between genotype and phenotype.

There are some limitations to this study. While DNA-M measurement using Illumina Infinium-HumanMethylation450 arrays has been shown to have reproducibility and high validity [24], technical replication of the DNA-M measurements has not been undertaken. We have not measured DNA-M in airway tissue; however, the major source of IL-13 production in the airways is inflammatory cells, and methylation measurements in peripheral blood are likely to better reflect asthma-related immune mechanisms. Cell composition in the peripheral blood can influence DNA-M; however, cell composition alone cannot explain the differential methylation observed [22]. Focusing only on female participants is a limitation; however, the results should be encouraging for further studies to replicate the model. The interaction effects seen may not imply a direct relationship between genotype and altered methylation. Genotype may also alter other epigenetic processes such as chromatin remodeling and this may then lead to altered DNA-M.

The effect of environment, genotype and DNA-M was seen for two different, but equally important and objective, characteristics of asthma: airflow limitation and AR. Similar to Michel and colleagues [22] we did not see an effect on asthma as an outcome (results not shown); however, the observed effect on asthma-related objective measures avoids plausible bias of reported asthma. Methylation of the promoter region can regulate gene transcription [25] and we have shown that promoter region methylation is dependent on genotype, and the interplay of the environment, genotype and DNA-M influence the phenotype.

While requiring replication in an independent cohort, the results show the interplay of prenatal maternal smoking, genetic variants and DNA-M of *IL13* influencing asthma-related lung function. This highlights the need to consider environment, genotype and DNA-M together when seeking to understand the pathogenesis of complex disease, as DNA-M plays a role of integrator of multiple disease pathway signals.

## Abbreviations

AR: Airway reactivity; CpG: Cytosine phosphate guanine; DNA-M: DNA methylation; DRS: Dose response slope; FEV1: Forced expiratory volume in 1 second; FVC: Forced vital capacity; IL: Interleukin; methQTL: Methylation, quantitative trait loci; modGV: Modifiable genetic variant; SNP: Single nucleotide polymorphism.

## Competing interests

The authors declare that they have no competing interests.

## Authors’ contributions

VKP contributed to design, analysis and wrote the manuscript draft. JWH contributed to design, interpretation of the data, manuscript preparation and review for intellectual content. HZ contributed to analysis, interpretation of the data and manuscript preparation. NSR contributed to data analysis and manuscript preparation. SE contributed to design, acquisition of data and revising the manuscript. SHA contributed to the concept, acquisition of data and review for intellectual content. WK contributed to the concept, design, analysis and review for intellectual content. All the authors have access to the data and have approved the manuscript version submitted.
